# Three-Dimensional Kinematics of the Pelvis and Caudal Lumbar Spine in German Shepherd Dogs

**DOI:** 10.3389/fvets.2021.709966

**Published:** 2021-08-26

**Authors:** Katharina I. Schaub, Nicola Kelleners, Martin J. Schmidt, Nele Eley, Martin S. Fischer

**Affiliations:** ^1^Department of Veterinary Clinical Sciences, Small Animal Clinic—Surgery, Justus-Liebig-University, Giessen, Germany; ^2^Department of Veterinary Clinical Sciences, Small Animal Clinic—Neurosurgery, Neuroradiology and Clinical Neurology, Justus-Liebig-University, Giessen, Germany; ^3^Institute of Zoology and Evolutionary Research, Friedrich-Schiller-University, Jena, Germany

**Keywords:** dog locomotion, scientific rotoscoping, lumbosacral motion, pelvic motion, three-dimensional kinematics

## Abstract

Lumbosacral vertebral motion is thought to be a factor in the development of degenerative lumbosacral stenosis in German shepherd dogs. So far, few studies exist describing natural canine lumbosacral movement *in vivo*. Therefore, this investigation aims to achieve a detailed *in vivo* analysis of bone movement of the lumbosacral region to gain a better understanding of the origin of degenerative lumbosacral stenosis using three-dimensional non-invasive *in vivo* analysis of canine pelvic and caudal lumbar motion (at L6 and L7). Biplanar cineradiography of the pelvis and caudal lumbar spine of four clinically sound German shepherd dogs at a walk and at a trot on a treadmill was recorded. Pelvic and intervertebral motion was virtually reconstructed and analyzed with scientific rotoscoping. The use of this technique made possible non-invasive measurement of physiological vertebral motion in dogs with high accuracy. Furthermore, the gait patterns of the dogs revealed a wide variation both between individual steps and between dogs. Pelvic motion showed a common basic pattern throughout the stride cycle. Motion at L6 and L7, except for sagittal rotation at a trot, was largely asynchronous with the stride cycle. Intervertebral motion in all dogs was small with approximately 2–3° rotation and translations of approximately 1–2 mm. The predominant motion of the pelvis was axial rotation at a walk, whereas lateral rotation was predominant at a trot. L7 showed a predominance of sagittal rotation (with up to 5.1° at a trot), whereas lateral rotation was the main component of the movement at L6 (about 2.3° in both gaits). During trotting, a coupling of various motions was detected: axial rotation of L7 and the pelvis was inverse and was coupled with craniocaudal translation of L7. In addition, a certain degree of compensation of abnormal pelvic movements during walking and trotting by the caudal lumbar spine was evident.

## Introduction

Diseases of the lumbosacral junction belong to the most common disorders of the musculoskeletal system in German shepherd dogs (GSDs) (1), which is why degenerative lumbosacral stenosis is a focus of clinical research. In dogs, degenerative lumbosacral stenosis (DLS) is known as the main cause of cauda equina syndrome (2). In DLS, degeneration and protrusion of the lumbosacral intervertebral disc and compression of the cauda equina nerves play an important role, with resulting pain and neurological failure (2–5). Large breed and working dogs seem to be particularly affected by DLS (2, 3); in fact, some authors have shown a breed predisposition for GSD, based on the particular morphology of the articular facets (6, 7). Despite many scientific studies, the true cause of DLS and the reasons for the predisposition of the GSD to it still remain uncertain. It was also stated that certain breeds, such as the GSD, are prone to premature intervertebral disc degeneration and DLS because of an abnormal movement pattern at the lumbosacral junction (5, 8, 9).

The complex motion of the canine lumbar spine has been the subject of several investigations, including range-of-motion studies on cadavers and kinematic studies using skin and bone markers. Each of these methods has certain limitations. Cadaver skeleton studies lack the influence of the surrounding soft tissue (8, 10–12). The highly invasive procedure of implanted bone markers is likely to interfere with natural movement (13, 14). Less invasive skin markers can only give an approximation of the motion of single vertebral bodies, due to the movement of the skin, which moves independently of the underlying skeletal elements (15, 16). One of the latest studies on canine lumbar kinematics was an investigation by Wachs examining lumbar and pelvic motion in three beagles by means of biplanar fluoroscopy and scientific rotoscoping (17). This method was also used in the current study due to its high measurement accuracy and low invasiveness.

The aim of the study was to perform a detailed, three-dimensional, non-invasive *in vivo* analysis of pelvic and caudal lumbar motion in healthy GSD at a walk and a trot. Furthermore, the study attempted to evaluate the benefit and accuracy of scientific rotoscoping, a markerless XROMM (X-ray Reconstruction of Moving Morphology) (18) method for the examination of canine lumbar vertebral kinematics.

## Materials and Methods

### Animals

Four healthy adult GSDs (one female, three male) of the working line were examined. The dogs had an average age of 22 ± 6 months, an average height of 61 ± 4 cm, an average weight of 34 ± 5 kg, and an average body condition score 4–5/9. All examined dogs came from private and breeding sectors and were active in sports.

### Ethics statement

The prospective part of the study was carried out in strict accordance with the recommendations in the Guidelines for the Care and Use of Laboratory Animals of the German Animal Protection Law. The protocol was approved by the Committee on the Ethics of Animal Experiments of the Justus Liebig University as well as from the Regierungspräsidium Hessen and Thuringia (Permit No.: 22-2684-04-02-075/14).

### Study Design

#### Part 1: Clinical Examination and Cross-Sectional Imaging

The dogs underwent a complete clinical workup including general, orthopedic, and neurologic examinations, to rule out diseases that could influence the gait or vertebral motion. Anesthesia was induced using an anesthetic protocol with diazepam (0.5 mg/kg i.v.) and xylazine (0.03 mg/kg i.v.) in combination with ketamine (3 mg/kg i.v.). Propofol (2–4 mg/kg i.v.) was used if needed. Anesthesia was maintained by isoflurane (1.5–3 vol%) in 100% oxygen.

CT images of the complete spine and pelvis were acquired using a 16-slice helical scanner (Brilliance Philips, Best, Netherlands) under general anesthesia, to gain individual morphological data. CT scan settings were 120 kV, 200 mA, and a slice thickness of 1 mm. In addition, an MRI scan of the spine was conducted to rule out an early stage of DLS. An Intera 1.0T^TM^ MRI scanner (Philips) was used in combination with the Syn-spine-coil. Sagittal T2-weighted images of the lumbar spine and transversal T2-weighted images at the level of L5–S1 were acquired. No dogs included in the study showed any signs of DLS at the time of the investigation.

#### Part 2: Treadmill-Assisted Biplanar Cineradiography and Gait Analysis

**Figure 1:**
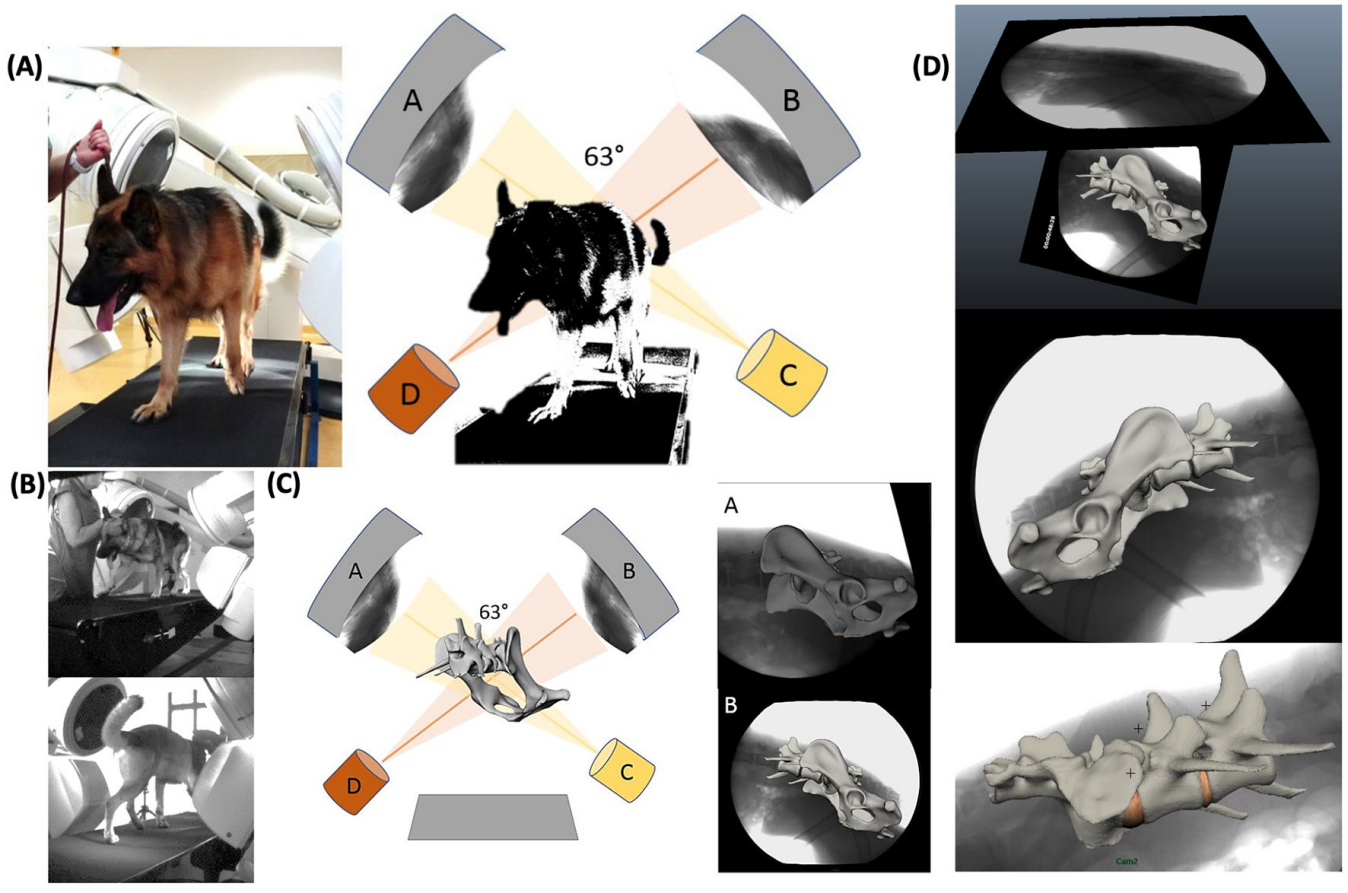
Experimental setup of the treadmill-assisted biplanar cineradiography and scientific rotoscoping with the image intensifiers set at 63°. **(A)** Physical experimental setup. X-ray films of lumbosacral motion are recorded in two different oblique lateral views (63°). **(A**, **B)** X-ray image intensifier with high-speed cameras. **(C**, **D)** X-ray tubes. **(B)** Livecam videos, recording motion synchronous to the X-ray films to correlate later motion curves with the stride cycle. **(C)** Scientific rotoscoping: virtual model of the real-life experimental setup in Autodesk Maya®. Schematical experimental setup with virtual bone marionette. **(D)** X-ray films with virtually adjusted bone marionette. Adjustment of the virtual bones in scientific rotoscoping is comparable to a shadow play—the virtual bone is rotated and slid until the bone silhouette is exactly congruent with both X-ray films.

The dogs were led on a horizontal mechanical treadmill at a walk and a trot. The speed of the treadmill depended on the comfort speed of the individual dog and was 0.8 ± 0.1 m/s for a walk and 2.4 ± 0.1 m/s for a trot. After an individual habituation time on the treadmill of about 10–20 min, biplanar X-ray high-speed videography (Neurostar Siemens AG, München and Visario Speedcam, Weinberger GmbH, Nürnberg) was performed. The biplanar X-ray videography system consisted of two C-arms with the treadmill in between. Depending on the size of the dog, the tube settings were 100 kV and 75 mA, and the shutter speed was set to 500 μs. The motion of the pelvis and caudal lumbar spine was recorded in two imaging planes at an angle of 63° at a walk and a trot for at least five steps. Simultaneously, the run was recorded with synchronous standard light high-speed live cameras (Standardlicht-Hochgeschwindigkeitkameras, Visario SpeedCam MiniVis®, High Speed Vision GmbH, Ettlingen, Germany) with 500 pics/s, to document the time when the feet rose and fell for evaluation of the duty factor and of disruptive movement (Figure 1 and Supplementary Videos 1, 2).

#### Part 3: Scientific Rotoscoping

Scientific rotoscoping is a non-invasive, markerless procedure of the XROMM method (18, 19) and a kinematic method to analyze natural skeletal motion in vertebrates *in vivo*. The detailed motion of the pelvis, sacrum, L6, and L7 was recorded using biplanar cineradiography. Based on individual CT scans of the spine and pelvis, a three-dimensional virtual bone marionette of the pelvis, sacrum, and the last two lumbar vertebrae was created, accurate in every detail, using the three-dimensional image processing program Amira 6® (Visage Imaging, Berlin, Germany). The experimental setup and the bone movement of the pelvis and vertebrae were virtually reconstructed by adjusting the virtual bone marionette to the biplanar X-ray videos using the graphic software Autodesk Maya 2014®. Since the two X-ray films were recorded from two different views, a moving three-dimensional virtual spine was created, virtually imitating the real three-dimensional bone movement with high precision (Figure 1 and Supplementary Videos 3, 4). Afterwards, three-dimensional motion measurements were performed using virtual animation. To minimize individual measurement inaccuracy, scientific rotoscoping was carried out by a single person (the first author, KS), and measurements were made after a period of training of about 12 weeks.

### Data Analysis

**Figure 2:**
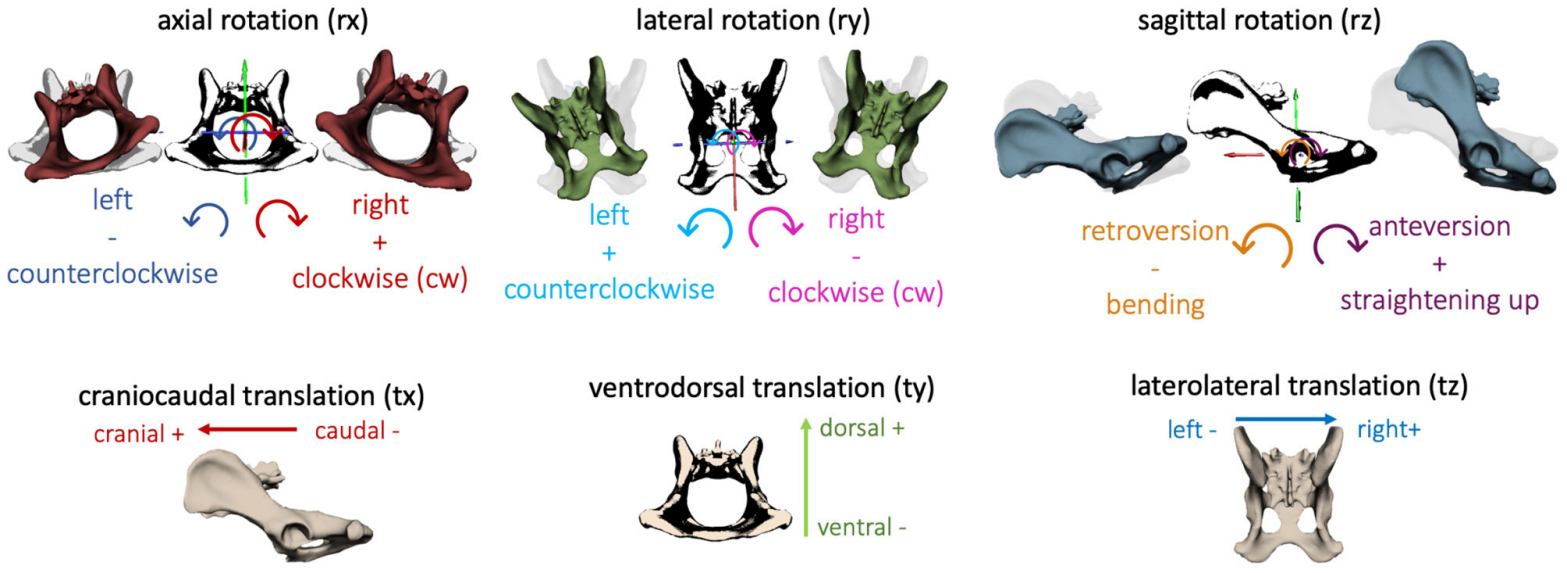
Definition of the direction of rotation and translation of the pelvis and vertebrae: axial rotation (rx)—rotation about the craniocaudal body axis, lateral rotation (ry)—rotation about ventrodorsal body axis, sagittal rotation (rz)—rotation about the laterolateral body axis; craniocaudal translation (tx), ventrodorsal translation (ty), laterolateral translation (tz).

In the present study, consecutive strides at a walk (*n* = 6) and a trot (*n* = 9) of the four participating GSDs were analyzed and described in six degrees of freedom. The directions of three-dimensional pelvic and vertebral movements were defined according to Wachs: axial rotation (rx) describing rotating movement around the craniocaudal body axis. Lateral rotation (ry) illustrated rotation around the ventrodorsal body axis, and sagittal rotation (rz) was defined as rotation around the laterolateral body axis (17). In addition, translational movement of the pelvis, L6, and L7 was analyzed based on craniocaudal translation (tx), ventrodorsal translation (ty), and laterolateral translation (tz) (Figure 2). Bone movement was described in relation to the adjoining caudal bone (17, 20).

To compare steps with various stance and swing phases, a stride normalization (21) of the individual steps was performed, based on the duty factor (22–24) using MATLAB (MATLAB®, The MathWorks, Massachusetts). The duty factor describes the percentage of the stance phase of a reference leg during the entire stride cycle. The duty factor of each dog was determined by means of gait analysis of the synchronized LifeCams. In the present study, the duty factor of the reference leg (left hindlimb; LH) was 0.7 ± 0.01 for a walk and 0.4 ± 0.01 for a trot (except for GSD 3: duty factor 0.5 ± 0.02).

Due to the minimal expected movements of the sacroiliac joint (25) and the overlap of the sacrum and pelvis in the X-ray videos, the sacrum was defined as a fixed connection with the pelvis. Therefore, in the following text, the motion of the pelvis simultaneously represents the movement of the sacrum.

Analyzed motion data included range of motion (ROM) of the facet joints and pelvis, time of occurrence (TOO) of maxima and minima within a stride cycle (15, 17), type of movement (dependent on the number of changes in motion direction), and dependence on the stride cycle. The collected data were compared between separate steps and individual dogs.

Data on pelvic and intervertebral motion were correlated between different steps, dogs, and anatomic locations using SPSS (SPSS®, Statistics for windows, Version 24.0, IBM Corp., Armonk, NY). Additionally, data were analyzed with the help of Fourier transformation with the command line interpreter Jupyter (Project Jupyter, www.jupyter.org). Due to the small number of patients and the high inter- and intraindividual variation in motion, even between individual steps of one dog, this study was limited to descriptive analysis. In the present study, a measurement accuracy of approximately 1.5° for rotation and approximately 0.1 cm for translational movements was achieved.

## Results

### Pelvic Rotation and Translation

**Figure 3:**
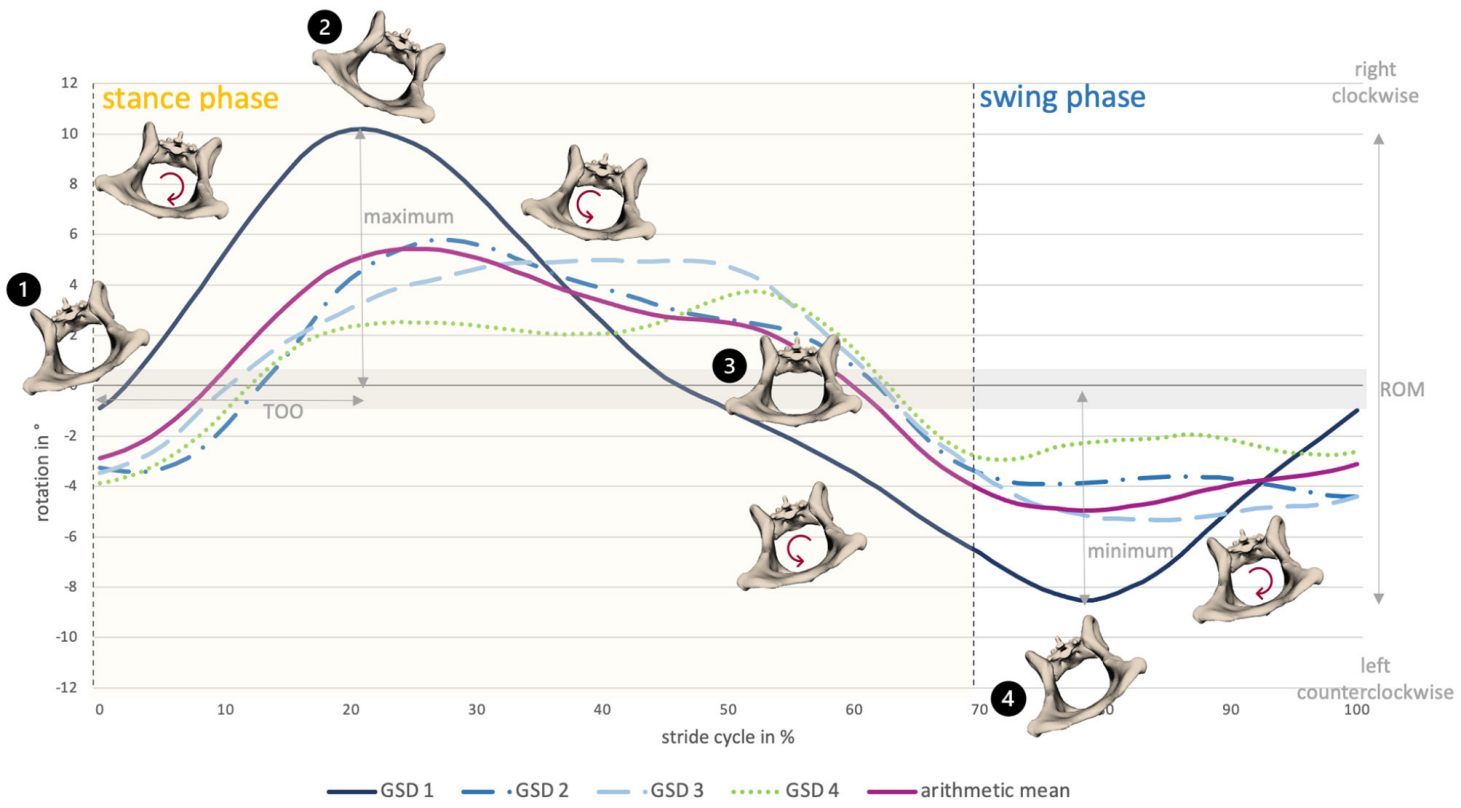
Pelvic axial rotation of all dogs at a walk. The anatomical diagrams visualize pelvic axial rotation of GSD 1. 1) Stride cycle starts with footing of the left hindlimb with the pelvis slightly tilted to the left, followed by a right-sided rotation. 2) Around the middle of the stance phase, the pelvis is maximally right tilted. 3) Afterwards, it rotates to the left side, reaching a neutral position shortly before the end of the stance phase. 4) Around the middle of the swing phase, the pelvis is maximally left tilted, then it rotates to the right side until the end of the swing phase.

Axial pelvic rotation (rx) demonstrated a monophasic motion pattern with one minimum and one maximum both at a walk and at a trot. For single steps of GSD 2, an intermittent bi- to triphasic motion was noticed at a trot, due to ipsilateral limb interactions. Motion was stride cycle-dependent and followed a basic pattern. In both gaits, the pelvis tilted in the direction of the foot touching the ground, starting with the left hindlimb (reference leg). During the stance phase, the pelvis rotated clockwise (caudocranial view) until the middle of the stance phase and then changed direction, rotating counterclockwise up to the middle of the swing phase; afterwards, it changed again to clockwise rotation until the end of the stride cycle (Figure 3).

Change in the direction of the motion occurred at a walk at 29.0 ± 9.0% and 86.8 ± 10.1% and at a trot at 33.0 ± 10.7% and 85.5 ± 18.6% of the stride cycle of the reference limb (Supplementry Table 1). In both gaits, intermittent, small elevations of the curve in terms of momentary changes in rotational direction were noticed. These were associated with the touch-down of a hind paw. Axial pelvic rotation showed the greatest ROM at a walk (12.1 ± 4.7°) in comparison with lateral and sagittal rotation. At a trot, ROM only reached values approximately 6.1 ± 5.7° (Supplementary Table 2).

**Figure 4:**
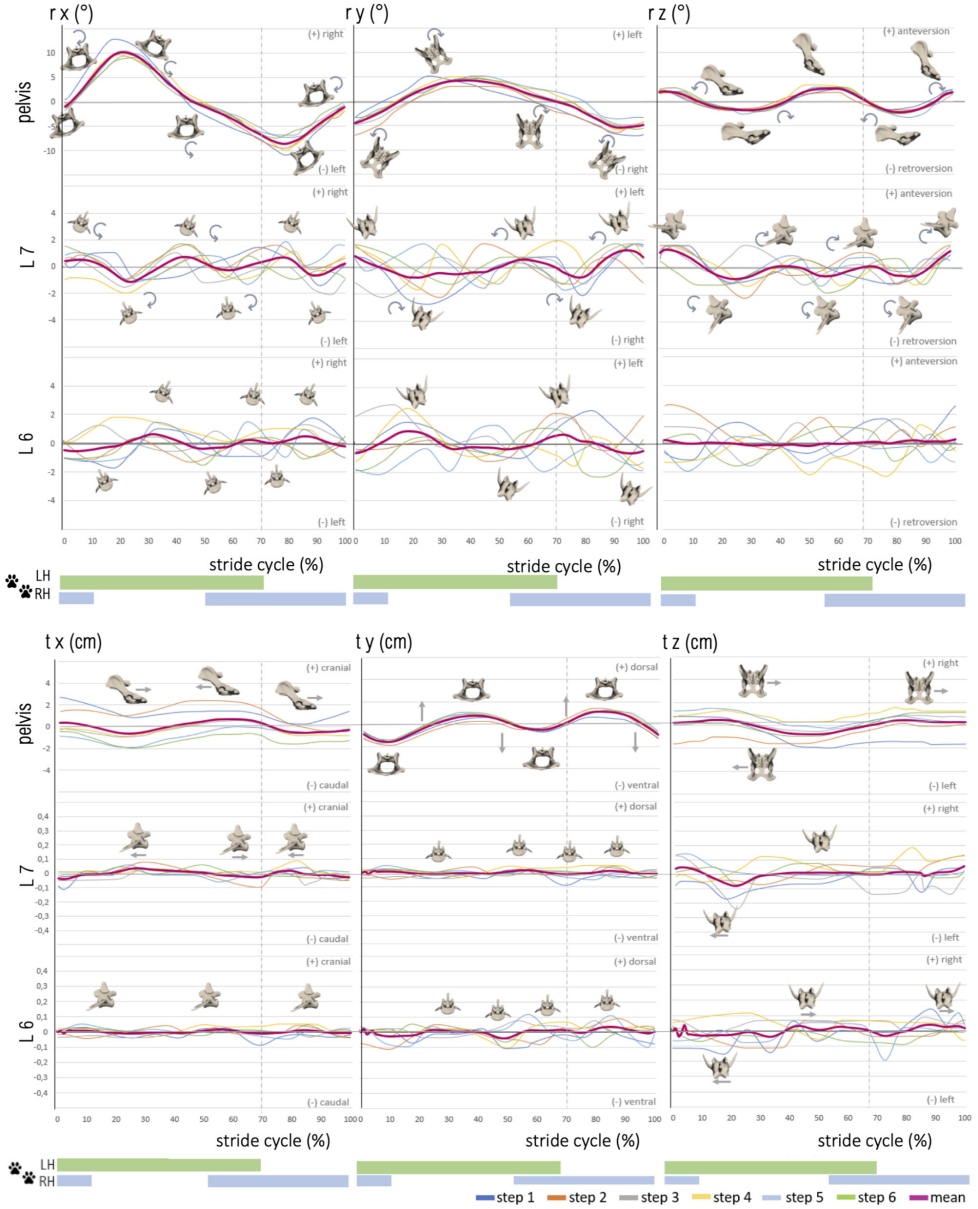
Stride phase-normalized stride cycle-dependent motion of pelvic and caudal lumbar vertebrae (L7, L6) of GSD 1 at a walk. Listed are motions of the separate bones in six degrees of freedom: rx (axial rotation), ry (laterolateral rotation), rz (sagittal rotation), tx (craniocaudal translation), ty (dorsoventral rotation), and tz (laterolateral translation). The different colored curves present the six analyzed individual steps of dog 1, dependent on the stride cycle [duty factor 0.7; LH, left hindlimb (reference leg); RH, right hindlimb]. The pink line represents the mean value graph of all six strides with anatomical diagrams symbolizing the stride cycle-dependent bone movement.

**Figure 5:**
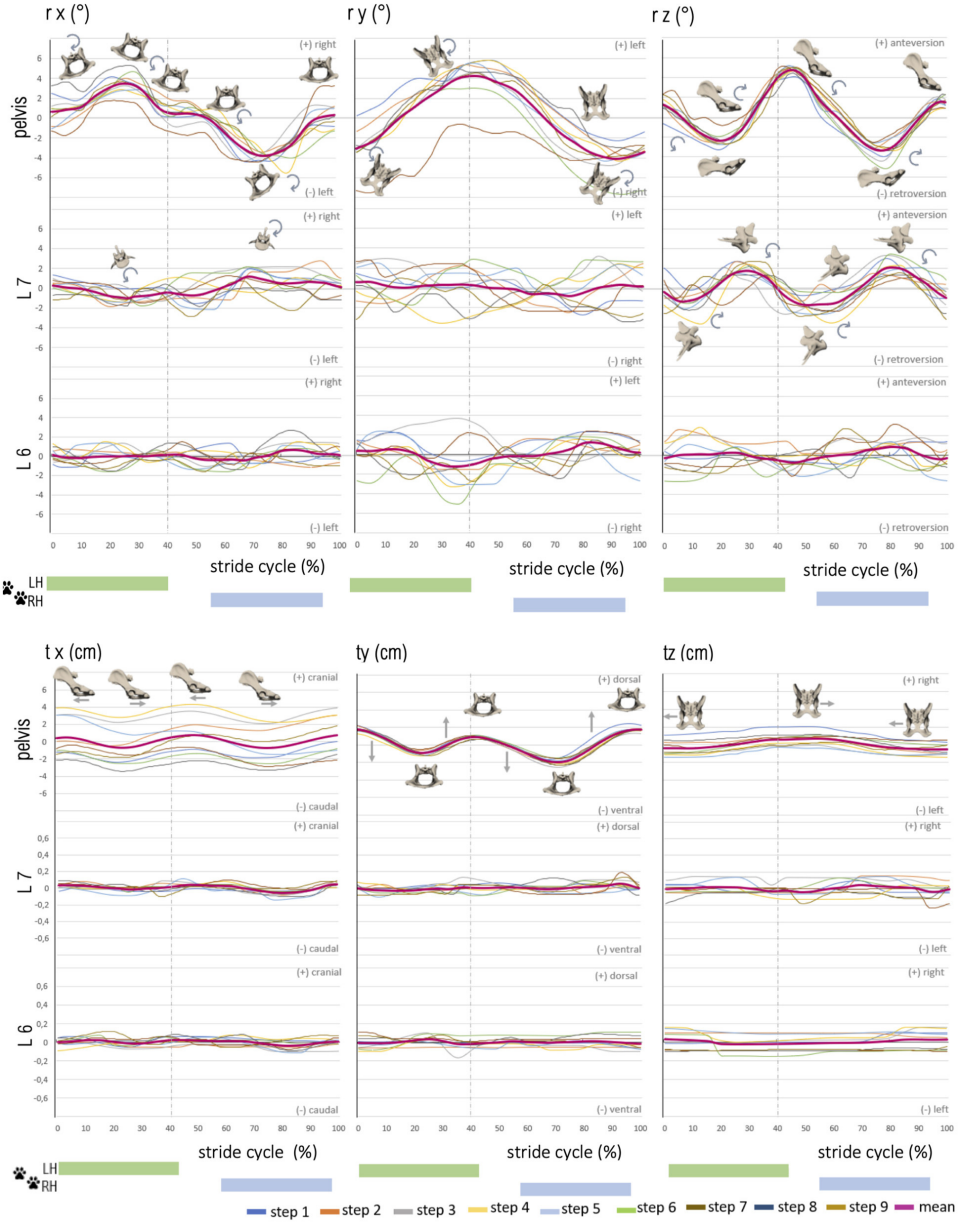
Stride phase-normalized stride cycle-dependent motion of pelvic and caudal lumbar vertebrae (L7, L6) motion of GSD 1 at a trot. Listed are motions of the separate bones in six degrees of freedom: rx (axial rotation), ry (laterolateral rotation), rz (sagittal rotation), tx (craniocaudal translation), ty (dorsoventral rotation), and tz (laterolateral translation). The different colored curves present the nine analyzed steps of dog 1, dependent on the stride cycle [duty factor 0.4; LH, left hindlimb (reference leg); RH, right hindlimb]. The pink line represents the mean value graph of all nine strides with anatomical diagrams symbolizing the stride cycle-dependent bone movement. Rotation and translation of L6 and partly L7 are widely asynchronous to the stride cycle with a high variety in TOO except for sagittal rotation of L7, and vertebral translation is minimal.

Lateral pelvic rotation (ry) was monophasic at a walk and a trot and followed a generally reproducible motion pattern (Figures 4, 5). A change in the direction of motion occurred at the mid to second half of the stance phase and approximately at touch-down of the hind paws in both gaits (walk: 43.3 ± 3.9% and 96 ± 4.0% of the stride cycle; trot: 39.3 ± 10.6% and 91.9 ± 8.3% of the stride cycle). ROM was similar at a walk and a trot: 10.9 ± 0.8° (walk) and 9.0 ± 0.9° (trot). Compared with axial and sagittal rotation, at a trot, lateral pelvic rotation reached the greatest values.

Sagittal pelvic rotation (rz) showed a biphasic motion at a walk and a trot (Figures 4, 5). Both maxima were associated with touch-down of the hindlimbs and represented maximal anteversion of the pelvis. Both minima occurred in mid stance and swing phase and expressed maximum retroversion of the pelvis (walk: maxima at 2.8 ± 1.5% and 54.3 ± 5.2%, minima at 30.6 ± 2.5% and 80.4 ± 3.6% of the stride cycle). ROM of the sagittal pelvis movement was 7.8 ± 2.5° for a walk and 6.7 ± 1.9° for a trot.

Translation of the pelvis was analyzed in the craniocaudal (tx), ventrodorsal (ty), and laterolateral (tz) directions (Figures 4, 5). Craniocaudal translation (tx) in the study was mainly influenced by the treadmill with a nearly linear curve during a walk and an implied biphasic curve during a trot. Maximal cranial translation occurred shortly after touch-down of the hindlimbs reflecting a strengthened cranial pelvic movement. ROM was 2.6 ± 0.6 cm (walk) and 2.9 ± 0.6 cm (trot) matching the constant forward movement of the dogs on the treadmill.

Pelvic ventrodorsal translation (ty) presented a biphasic motion in both gaits with maxima at 3.6 ± 4.0% and 81.2 ± 4.3% (walk) and 43.5 ± 3.5% and 93.2 ± 6.2% (trot) of the stride cycle. Minima were noted at 7.2 ± 2.8% and 57.4 ± 3.2% (walk) and 17.5 ± 5.2% and 70.5 ± 5.3% (trot) of the stride cycle. ROM of ventrodorsal pelvis translation was similar in both gaits with 3.6 ± 0.8° (walk) and 3.6 ± 0.6° (trot). Laterolateral translation (tz) of the pelvis showed a nearly linear movement with ROM of 3.5 ± 1.6 cm (walk) and 2.6 ± 0.7 cm (trot) in both gaits and suggested a steady position on the treadmill.

### Intervertebral Motion of L6 and L7

Intervertebral motion was measured at the level of the facet joints of L6–L7 and L7–S1. ROM was in most cases approximately 2–3° at the level of L6 and L7, whereas lateral rotation was the dominant motion at L6 [~3.4° (walk) and 3.8° (trot)]. At L7, sagittal rotation achieved the greatest ROM in both gaits, with ~5.1° while trotting. Axial intervertebral rotation (rx) (Figures 4, 5) of L7 was partly biphasic and partly triphasic at a walk. No common stride dependency was found. Instead, a certain negative dependence of axial rotation of L7 and the pelvis was noted. ROM was similar in both gaits (walk: 3.0 ± 0.5°, trot: 3.0 ± 0.3°).

At the level of L6, no reproducible gait pattern-related motion could be found between the steps of one dog nor between those of different dogs. This was also reflected in the high standard deviations of the individual steps. ROM at the level of L6 was only slightly smaller than the lumbosacral ROM (walk: 2.5 ± 0.4°, trot: 2.3 ± 0.2°).

Lateral intervertebral rotation (ry) of L7 had a similar magnitude in both gaits, with a ROM of 3.6 ± 0.6° (walk) and 3.4 ± 1.0° (trot) and showed no gait–cycle relation. Whereas a bi-to-triphasic motion was seen in most dogs at a walk, lateral rotation of L7 at a trot was irregular biphasic (Figures 4, 5). The motion pattern between the separate steps of one dog was inconstant and showed great variation, noticeable in the high standard deviation of the TOO. Interindividual variation between dogs was smaller than intraindividual variation between the steps of one dog. Lateral rotation of L7 differed clearly from lateral pelvic movement.

Lateral rotation of L6 was neither related to the TOO of L7 nor to pelvic rotation or stride cycle. The TOO of L6 differed greatly between dogs and between steps of individual dogs. ROM of L6 was 3.4 ± 1.0° at a walk and 3.9 ± 0.4° at a trot. Lateral rotation was, at the level of L6, the dominating rotational motion in both gaits.

Sagittal intervertebral rotation (rz) at L6 and L7 at a walk was only mildly above the measurement limit. A uniform motion pattern with a greater ROM was only detected at a trot (Figures 4, 5).

Sagittal rotation of L7 was irregular, biphasic, and sometimes triphasic at a walk, without any reproducible motion pattern. The TOO between separate steps and individual dogs demonstrated great variability. ROM was 3.6 ± 0.7° at a walk. At a trot, however, sagittal rotation of L7 in all dogs showed a strong dependence on the stride cycle with a reproducible motion pattern. GSD 1, 2, and 4 demonstrated a nearly concurrent motion pattern, whereas dog 3 had a similar motion pattern but with a delayed occurrence of the first maximum. TOO for sagittal intervertebral rotation of L7 for the maxima was 30.0 ± 5.7% and 83.8 ± 1.4%, and for the minima, it was 9.6 ± 1.5% and 57.7 ± 4.0% of the stride cycle. ROM was 5.1 ± 0.5° and, therefore, the dominant rotational motion at the level of L7.

At L6, at a walk, no dependence of sagittal intervertebral rotation on the stride cycle was detected. ROM reached a maximum level of 3.1 ± 0.9°. At a trot, a biphasic motion pattern with ROM of 3.3 ± 0.3° was noted. In comparison with L7, the motion of L6 was much more irregular. The TOO was less synchronic in a step pattern of singular steps and different dogs (maxima: 22.4 ± 5.9%, 70.7 ± 7.1%; minima: 36.3 ± 6.4%, 80.3 ± 10.9%).

Intervertebral translation of L6 and L7 was only minimal, 0.1–0.2 cm in all directions, and thus at the resolving limit of the research method. A reproducible biphasic stride cycle-dependent motion pattern was only noticed at a trot for craniocaudal translation of L7, with maxima at 6.0 ± 3.9% and 55.3 ± 5.5% and minima at 31.8 ± 4.5% and 81.5 ± 2.7% of the stride cycle (Figures 4, 5).

### General Lumbosacral Motion

**Figure 6:**
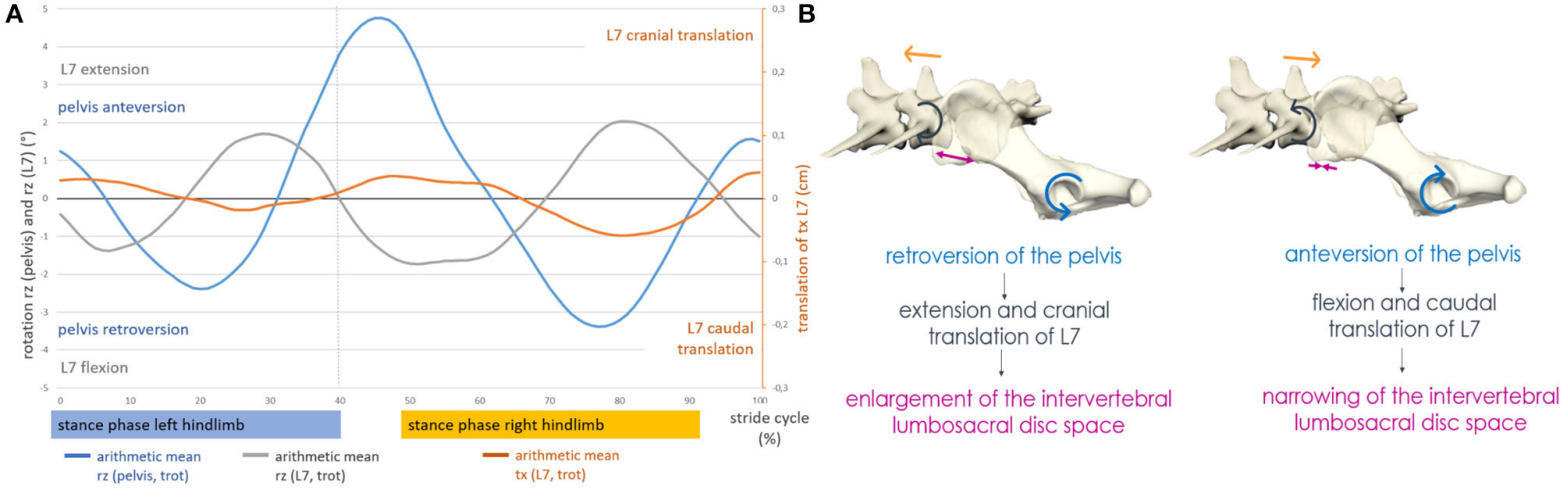
**(A)** Motion of the lumbosacral junction at a trot of GSD 1: coupled motion of craniocaudal translation of L7 and sagittal rotation of the pelvis and L7. **(B)** Schematic illustration of the lumbosacral movement in GSDs during trotting. Lumbosacral motion in GSDs is complex and comparable with the motion of train couplers. The lumbosacral facet joints serve as a hinge, allowing deformation of the intervertebral disc space into a reverse V shape in case of retroversion of the pelvis. In case of anteversion of the pelvis, the hinge formed by the facet joints will only allow narrowing of the intervertebral disc space in the ventral aspect but prevent widening in the dorsal aspect by the limited translation within the facet joints.

Axial pelvic rotation and axial rotation of L7 were directed inverse. L6 followed the axial rotation of L7, but with a mild delay and a phase shift. Deviations in pelvic motion, resulting from ipsilateral limb interaction, flexion of the spine, or displacement of the caudal body axis, affected the motion of the caudal lumbar spine. In the case of deviating axial pelvic rotation, the caudal lumbar vertebrae showed a forced axial rotation in the opposite direction, although the basic motion remained and was only mildly affected. This context was also noticed in lateral and sagittal rotation but was less pronounced. Therefore, variant pelvic and hindlimb motions were compensated to a certain degree by opposing directed movements of the caudal lumbar spine (mostly L7 and sometimes L6) as well as by subsequent pelvic movement. When the lumbosacral motion was examined, an inverse sagittal rotation between the pelvis and L7 was observed, but only at a trot. In addition, at a trot, coupling of craniocaudal translation to sagittal rotation of L7 was evident (Figure 6).

Motion data were correlated using Spearman's correlation, with solitary bones, strides, and individual dogs to verify a common motion pattern. Pelvic rotation showed a strong correlation in all dogs and strides. In pelvic translation, only ventrodorsal translation reached high correlation values. L6 and L7 showed a mild correlation for rotation and translation between strides. Only sagittal rotation of L7 showed a moderate stride correlation. Motion patterns between different dogs were more strongly correlated and thus more consistent than different strides of one dog. However, except for pelvic rotation, correlation was mild to moderate between dogs. Rotation of L6 and L7 was negatively correlated with pelvic rotation, above all at a trot.

**Figure 7:**
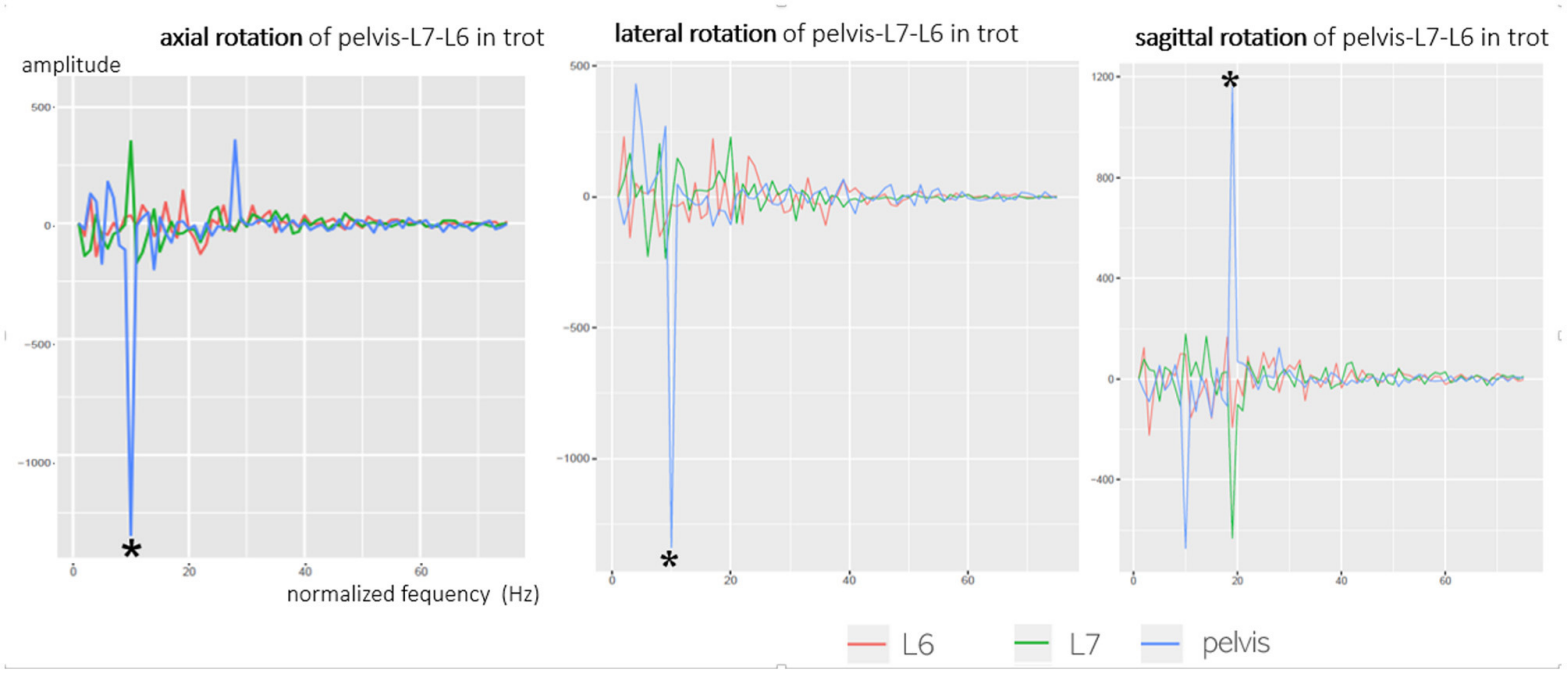
Fourier transformation (cosine part) of axial, lateral, and sagittal rotation of the pelvis, L6, and L7, exemplary GSD 2 at a trot. Axial rotation shows one main frequency (*) and many smaller spurious oscillations. The main oscillations (*) of the axial rotation of the pelvis (blue line) are directed opposite to L6 (red line) and L7 (green line) as the pelvis shows a negative amplitude, whereas L6 and L7 show a positive amplitude. This means that the pelvis and caudal lumbar spine show an inverse axial oscillation.

Fourier transformation breaks down complex motion curves into their underlying frequencies for a better investigation of linkages. Peaks in the Fourier transformation indicate similar reproducible strides. A comparison of axial rotation of the pelvis, L6, and L7 confirmed the described observations: the main oscillation of axial rotation was based on the cosine function with one main frequency and multiple secondary frequencies in a different manner (Figure 7). At all main oscillations of the pelvis, an opposite direction of the vibration of L6 and L7 was proven, which also matches the negative pelvic–L7/L6 correlation. Fourier transformation verified that the pelvis and caudal lumbar spine oscillated inversely in the axial direction. The same context was found concerning sagittal rotation of the pelvis and L7/L6; however, this was only observed at a trot. While pelvic oscillations were similar in all dogs and had a clear main oscillation, oscillations of L6 and L7 had a relatively wide frequency spectrum and differed between individual dogs. Fourier analysis suggests that rotational motion of L6 and L7 is not only dependent on pelvic movement but also influenced by other motion factors—this fits with the significant inhomogeneity of the intervertebral rotation curves.

## Discussion

To the authors' knowledge, this is the first study providing a detailed insight into natural lumbosacral motion in GSD. To date, a similar investigation of lumbosacral kinematics has only been performed in three beagles, also using scientific rotoscoping (17).

Scientific rotoscoping offers outstanding measurement accuracy for investigations of canine lumbosacral motion *in vivo*, along with minimal invasiveness. However, even this method reaches the limits of spatial resolution when defining exceedingly small intervertebral movements. Despite the many advantages of scientific rotoscoping, it is a very time-consuming and complicated process. Therefore, only small numbers of patients can be examined with scientific rotoscoping at the moment. Thus, the method is reserved for research and is currently unsuitable for clinical examination due to the high cost and time requirement.

Pelvic axial and lateral rotation were monophasic in both gaits, whereas pelvic sagittal rotation revealed biphasic motion at a walk and a trot. Pelvic rotation showed strong stride cycle dependence and displayed a reproducible motion pattern with only mild individual variation. These observations are mostly consistent with studies conducted on beagles (17). The main motion of the caudal lumbar spine originated from the pelvis and, for the most part, followed pelvic motion passively. Isolated intervertebral motion on its own, however, was small. Pelvic rotation was mainly a result of hindlimb movement and thus showed a strong dependence on the stride cycle. As in research conducted on beagles, pelvic motion displayed a common reproducible basic gait pattern in all GSDs, which was, however, subject to individual variation (17).

In the present study, axial pelvic rotation dominated with 12.1 ± 4.7° at a walk, whereas at a trot, lateral rotation (ROM 9.0 ± 0.9°) was the predominant motion. In GSD, motion of the hindlimbs was mainly transferred to the spine as propulsion in trot, as lateral pelvic rotation dominated here. This is in contrast to studies on horses (26, 27) and beagles (17). The GSD, beagle, and horse showed similar pelvic axial rotation ROM at a walk, whereas the beagle showed a higher ROM (10.9 ± 0.8°) at a trot than the GSD (6.1 ± 5.7°) and horse (5.7 ± 0.9°) (17, 26). Only GSD 3 showed a similar ROM to the beagle; both the beagle and GSD 3 showed an identical duty factor of 0.5 at a trot. A certain dependency of axial pelvic rotation on the duty factor is therefore likely.

Lateral pelvic rotation was monophasic and stride cycle-dependent with maxima and minima associated with the footing of the hindlimbs. This fits with the observations on beagle (17). ROM was only slightly greater at a walk than at a trot (10.9 ± 0.8 vs. 9.0 ± 0.9°) and was the dominant pelvic rotation at a trot. GSDs showed greater lateral pelvic rotation than horses and beagles (beagle 4.6 ± 1.1° walk/5.8° trot vs. horse 5.1 ± 1.7° walk/4.1 ± 1.0° trot) (17, 27, 28). The aforementioned results indicate that in GSD, hindlimb movement is mainly transferred to propulsion at a trot.

Sagittal pelvic rotation was biphasic in both gaits and stride cycle-dependent, as in previous studies in dogs (16, 17). In GSD, a ROM of 7.8 ± 2.5° at a walk and 6.7 ± 1.9° at a trot was documented for sagittal pelvic rotation. At a walk, similar values are known for beagles (approximately 8°) (17) and horses (~7.3 ± 1.4°) (27). At a trot, however, sagittal pelvic rotation varied slightly at 8.2 ± 1.0° in beagles and at 4.3° in horses (17, 28). In GSD, sagittal pelvic rotation was the smallest at a walk and only slightly greater than axial rotation at a trot, probably resulting from muscular stabilization of the pelvis and sacrum in the sagittal plane (M. longissimus and M. multifidus) (29, 30).

Craniocaudal and laterolateral pelvic translation was mainly dependent on the position of the dog on the treadmill. Ventrodorsal pelvic translation was biphasic and described the up and down movement of the pelvis, caused by the hindlimbs pushing off the ground; ROM was similar at a walk and a trot, resulting from a relatively constantly maintained pelvic position despite larger, bouncy steps at a trot. This supports the thesis that in GSD, hindlimb motion is mainly transferred to propulsion of the trunk.

Only small intervertebral motion was detected in the caudal lumbar spine. ROM of intervertebral rotation at L6 and L7 reached ~2–3° for rotation and approximately 1–2 mm for translation. This coincides with observations on the beagle that the main movement of the caudal lumbar spine originates from the pelvis (17). Nevertheless, this result contradicts the description of canine cadaver studies, in which sagittal rotation of up to 39° was observed (8). Thus, in natural motion of living dogs, only a fraction of the possible ROM is used in the symmetrical gaits at the lumbosacral junction. This supports the assumption of a stabilizing effect of the epaxial muscles on the spine (17, 30). In GSD, sagittal rotation was the predominant motion at the lumbosacral junction, with up to 5.1 ± 0.5° at a trot, while the lateral rotation was greatest in L6 at ~3.8° trot at both a walk and a trot. This differs from observations on the beagle, where axial rotation was the dominant direction of movement at the lumbosacral junction (beagle: walk 3.8 ± 0.6°/trot 4.9 ± 0.4°) (17).

When the lumbosacral motion was examined, an inverse sagittal rotation between L7 and the pelvis was noted during trotting. This context was also described in the beagle (17). Furthermore, a coupling of craniocaudal translation and sagittal rotation of L7 was observed in all GSDs. However, this context was only found at a trot. During this motion coupling, retroflection of the pelvis caused simultaneous extension and cranial translation of L7, resulting in a widening of the lumbosacral intervertebral disc space. By contrast, anteversion of the pelvis during trot led to flexion and simultaneous caudal translation of L7 and thus resulted in increased narrowing of the lumbosacral intervertebral disc space. The widening and narrowing of the intervertebral lumbosacral disc space occurred twice per stride cycle. This context was already described in a canine cadaver study and was now confirmed for the first time in living dogs during natural locomotion (8). It should be noted, however, that in the present study, this motion coupling at the lumbosacral junction was only observed at a trot.

When pelvic and lumbar rotation was examined, it was conspicuous that deviations in pelvic movement affected the caudal lumbar spine to a certain degree. When the pelvis, for example, showed an increased negative (right-sided) lateral rotation, L7 simultaneously performed an increased movement in the opposite direction. This coherence was already described in dogs with lameness of one hindlimb that presented with an increased longitudinal axis of rotation from the back to the healthy side (31). In the present study, it can be assumed that even small deviations in pelvic movement caused, for example, by tripping or turning are compensated by the caudal lumbar spine to some extent and thus contribute to the balance of the trunk. This supports the assumption of Wachs that extreme movements during sports put increased strain on the lumbosacral intervertebral disc (29).

Besides proving an inverse axial and sagittal rotation of the pelvis and L7, Fourier transformation also showed that axial and sagittal rotation of the caudal lumbar spine consist of one main oscillation and many smaller secondary frequencies. It can be assumed that the significant main frequency of axial vertebral rotation in Fourier translation represents the monophasic main motion of the pelvis and caudal lumbar spine, induced by motion of the hindlimbs. Pelvic axial rotation showed a clear main frequency, whereas the main frequency of the caudal lumbar spine differed by much less from the secondary frequencies. This suggests that compared with pelvic axial rotation, axial rotation of L6 and L7 is more influenced by other movements. Based on synchronous live videos, an impact of tail motion and hindlimb touch-down seemed likely in the present study. A similar hypothesis was stated in a gait analysis of Labrador retrievers and Dachshunds, where a superposition of monophasic caudal lumbar and sacral motion with motion of the tail and resulting oscillation interferences was suspected (16). For further evaluation, a combination of scientific rotoscoping with skin markers would improve the evaluation of this context.

Both pelvic and intervertebral motion presented wide variation between dogs and between strides in both gaits. While pelvic motion was relatively stereotypical in individual dogs and followed the same basic gait pattern, a basic pattern was hardly recognized in intervertebral motion, except for sagittal rotation at a trot. Pelvic motion differed above all between dogs, whereas intervertebral motion showed more similarities between dogs than between strides of one dog. Therefore, every stride, also within one dog, is unique, even if it follows the same basic motion pattern (23, 32). As every single step is influenced by several different factors (33), this explains the variation in intervertebral motion in the present study. Therefore, gaining reference values for kinematic gait analysis and lumbosacral motion is challenging, even in healthy dogs of a single breed. It can be assumed that this singularity of movement will play a central role in answering diagnostic questions in the future.

In the current literature, the canine lumbosacral junction is described as a spinal section of high mobility (8, 11, 34). The vertebral disc ensures stability between adjacent vertebrae (35–38), and therefore, lumbosacral disc degeneration seems to play an important role in the origin of canine DLS (2, 4, 5, 35). An increased lumbosacral vertebral translation, like the often-seen spondylolisthesis in X-rays (2, 4–6), an altered ROM, and an abnormal motion type are discussed as causes of DLS in GSDs (2, 4, 5, 11). This theory could not be confirmed in the present study, as all GSDs showed only minimal intervertebral translation and rotation in the caudal spine, comparable with those seen in the beagle (17). In GSD, sagittal rotation is the predominant motion at the lumbosacral junction, above all at a trot. The described coupling of sagittal rotation with craniocaudal translation increases the stretching and compression of the lumbosacral intervertebral disc during trotting. It can be assumed that the lumbosacral intervertebral disc in the GSD is exposed not only to repeated stress in the context of axial rotation, especially during trotting, but also to high stress from sagittal compression forces. A possible interpretation would be that this strong lumbosacral sagittal motion in GSD with compression of the intervertebral disc, in combination with the repeated shear movements as part of the axial rotation of L7, puts increased strain on the lumbosacral intervertebral disc, especially during trotting, thus contributing, in addition to extreme movements, for example, in sports, to early disc degeneration in this breed.

In GSDs, lumbosacral transmission of hindlimb and pelvic motion on L7 differs significantly from that in the beagle (17). Altered motion transmission in the GSD might be a consequence of the precipitous and flat lumbosacral facet joint geometry (9), making sagittal rotation the main lumbosacral motion component at a trot. It can be assumed that sagittal rotation is still sufficiently stabilized at a walk by the epaxial muscles resulting in a small ROM (29, 30, 39). At a trot, however, high lumbosacral sagittal rotation of GSDs suggests that muscular stabilization at the lumbosacral transition in faster gaits is no longer sufficient or has been reduced in favor of propulsion. The special lumbosacral motion transmission in the GSD in comparison with that in other breeds such as the beagle (17) indicates an increased strain on the lumbosacral intervertebral disc, probably resulting in early disc degeneration. An increased ventrodorsal vertebral translation, often associated with DLS, could not be observed in the GSDs in the present study. Therefore, it is possible that increased ventrodorsal lumbosacral translation in GSDs is not the cause but rather the consequence of lumbosacral disc degeneration in DLS and therefore could not be observed in the healthy dogs of the study (37, 38, 40, 41).

The distinct lumbosacral sagittal rotation combined with dominant pelvic lateral rotation at a trot suggests an optimal and effective propulsive motion transmission of hindlimb locomotion on the caudal lumbar spine in the GSD as a trotter (FCI guidelines). Probably, this effective propulsive lumbosacral motion transmission is associated with the special facet joint anatomy of GSD (9) but unfortunately seems to be at the expense of the lumbosacral vertebral disc. To further investigate this hypothesis, larger-scale studies of different dog breeds regarding lumbosacral *in vivo* kinematics and facet joint geometry are necessary.

## Conclusion

The present study demonstrates that a non-invasive measurement of physiological vertebral motion in dogs was possible with high accuracy, by means of scientific rotoscoping. Additionally, it has become evident that the canine gait pattern shows great diversity and varies widely both between individual dogs and between strides. Pelvic motion was based on a fundamental gait pattern, depending on hindlimb locomotion. Caudal lumbar motion was largely asynchronous with the stride cycle and showed high variation in dogs and strides, except for sagittal rotation at a trot. The main motion of the caudal lumbar spine originated from the pelvis, whereas isolated intervertebral caudal lumbar motion was small with approximately 2–3° rotational and ~1–2 mm translational ROM. Consequently, intervertebral motion is influenced by more factors than solely the movement of the hindlimbs.

The main direction of motion differed depending on the location. In pelvic motion, axial rotation was the dominant component at a walk, whereas lateral rotation was predominant at a trot. At L7, sagittal rotation was the highest (with up to 5.1° at a trot), whereas lateral rotation was the main component of the movement at L6. At a trot, coupling of various motions was detected. Sagittal rotation of the pelvis and L7 was directed inverse and presented coupling with craniocaudal translation. In addition, a compensation of abnormal pelvic movements by L7 and partially by L6 was demonstrated.

The study provides a first detailed insight into *in vivo* kinematics of the lumbosacral junction of GSDs, provides a basis for further comparative studies on other breeds and dogs with DLS, and therefore contributes to a better understanding of the cauda equina syndrome in GSDs.

## Data Availability Statement

The raw data supporting the conclusions of this article will be made available by the authors, without undue reservation.

## Ethics Statement

The animal study was reviewed and approved by the Committee on the Ethics of Animal Experiments of the Justus Liebig University as well as from the Regierungspräsidium Hessen and Thuringia (Permit No.: 22-2684-04-02-075/14). Written informed consent was obtained from the owners for the participation of their animals in this study.

## Author Contributions

KS, NK, NE, MS, and MF conceived the study. KS and NK conducted the experiments. KS prepared the X-ray data, generated the digital bone models from animal data, performed the rotoscoping, and analyzed the experimental data. KS, MS, and MF drafted the manuscript. All authors contributed to the interpretation of the results and revised the manuscript.

## Conflict of Interest

The authors declare that the research was conducted in the absence of any commercial or financial relationships that could be construed as a potential conflict of interest.

## Publisher's Note

All claims expressed in this article are solely those of the authors and do not necessarily represent those of their affiliated organizations, or those of the publisher, the editors and the reviewers. Any product that may be evaluated in this article, or claim that may be made by its manufacturer, is not guaranteed or endorsed by the publisher.

## Abbreviations

DF: duty factor
FCI: federation cynologique internationale
GSD: German shepherd dog
L6: sixth lumbar vertebra
L7: seventh lumbar vertebra
LH: left hindlimb (reference leg)
ROM: range of motion
rx: axial rotation
ry: lateral rotation
rz: sagittal rotation
S1: first sacral vertebra
TOO: time of occurrence
tx: craniocaudal translation
ty: ventrodorsal translation
tz: laterolateral translation
XROMM: X-ray reconstruction of moving morphology.

## References

[1] Vilson Å Bonnett B Hansson-Hamlin H Hedhammar Å. Disease patterns in 32,486 insured German shepherd dogs in Sweden: 1995–2006. Vet Rec. (2013) 5:116. 10.1136/vr.101577 23812178

[2] Suwankong N Meij BP Voorhout G de Boer AH Hazewinkel HA. Review and retrospective analysis of degenerative lumbosacral stenosis in 156 dogs treated by dorsal laminectomy. Vet Comp Orthop Traumatol. (2008) 3:285–93. 10.1055/s-0037-1617374 18536857

[3] Danielsson F Sjöström L. Surgical treatment of degenerative lumbosacral stenosis in dogs. Vet Surg. (1999) 2:91–8. 10.1053/jvet.1999.0091 10100762

[4] Oliver JE Jr Selcer RR Simpson S. Cauda equina compression from lumbosacral malarticulation and malformation in the dog. J Am Vet Med Assoc. (1978) 2:207–14. 681230

[5] Jaggy A Lang J Schawalder P. Cauda equina-Syndrom beim Hund [Cauda equina syndrome in the dog]. Schweiz Arch Tierheilkd. (1987) 4:171–92. 3589637

[6] Amort KH Ondreka N Rudorf H Stock KF Distl O Tellhelm B. MR-imaging of lumbosacral intervertebral disc degeneration in clinically sound German shepherd dogs compared to other breeds. Vet Radiol Ultrasound. (2012) 3:289–95. 10.1111/j.1740-8261.2011.01903.x 22372662

[7] Flückiger MA Damur-Djuric N Hässig M Morgan JP Steffen F. A lumbosacral transitional vertebra in the dog predisposes to cauda equina syndrome. Vet Radiol Ultrasound. (2006) 1:39–44. 10.1111/j.1740-8261.2005.00103.x 16429983

[8] Benninger MI Seiler GS Robinson LE Ferguson SJ Bonél HM Busato AR. Three-dimensional motion pattern of the caudal lumbar and lumbosacral portions of the vertebral column of dogs. Am J Vet Res. (2004) 5:544–51. 10.2460/ajvr.2004.65.544 15141871

[9] Breit S Künzel W. On biomechanical properties of the sacroiliac joint in purebred dogs. Ann Anat. (2001) 2:145–50. 10.1016/S0940-9602(01)80036-4 11325061

[10] Bürger R Lang J. Kinetische Studie über die Lendenwirbelsäule und den lumbosakralen Übergang beim Deutschen Schäferhund. Teil 1: Funktionelle Anatomie und kinetische Grundlagen [Kinetic study of the lumbar vertebrae and the lumbosacral passage in German shepherd dogs. 1. Functional anatomy and kinetic foundation]. Schweiz Arch Tierheilkd. (1992) 9:411–6. 1455213

[11] Bürger R Lang J. Kinetische Studie über die Lendenwirbelsäule und den lumbosakralen Ubergang beim Deutschen Schäferhund. Teil 2: Eigene Untersuchungen [Kinetic studies of the lumbar vertebrae and the lumbosacral transition in the German shepherd dog. 2. Our personal investigations]. Schweiz Arch Tierheilkd. (1993) 2:35–43. 8456269

[12] Benninger MI Seiler GS Robinson LE Ferguson SJ Bonél HM Busato AR Lang J. Effects of anatomic conformation on three-dimensional motion of the caudal lumbar and lumbosacral portions of the vertebral column of dogs. Am J Vet Res. (2006) 1:43–50. 10.2460/ajvr.67.1.43 16426210

[13] Wood KB Schendel MJ Pashman RS Buttermann GR Lewis JL Ogilvie JW Bradford DS. In vivo analysis of canine intervertebral and facet motion. Spine. (1976) 10:1180–6. 10.1097/00007632-199210000-00009 1440007

[14] Schendel MJ Dekutoski MB Ogilvie JW Olsewski JM Wallace LJ. Kinematics of the canine lumbar intervertebral joint. An in vivo study before and after adjacent instrumentation. Spine. (1995) 23:2555–64. 10.1097/00007632-199512000-00015 8610250

[15] Gradner G Bockstahler B Peham C Henninger W Podbregar I. Kinematic study of back movement in clinically sound malinois dogs with consideration of the effect of radiographic changes in the lumbosacral junction. Vet Surg. (2007) 5:472–81. 10.1111/j.1532-950X.2007.00294.x 17614929

[16] Layer AF. Ganganalytische Untersuchung der Rückenbewegung von gesunden Hunden der Rassen Dackel und Labrador Retriever (doctoral thesis). Ludwig Maximilian University of Munich, Munich, Germany (2012).

[17] Wachs K Fischer MS Schilling N. Three-dimensional movements of the pelvis and the lumbar intervertebral joints in walking and trotting dogs. Vet J. (2016) 210:46–55. 10.1016/j.tvjl.2015.12.009 26831181

[18] Gatesy SM Baier DB Jenkins FA Dial KP. Scientific rotoscoping: a morphology-based method of 3-D motion analysis and visualization. J Exp Zool A Ecol Genet Physiol. (2010) 5:244–61. 10.1002/jez.588 20084664

[19] Brainerd EL Baier DB Gatesy SM Hedrick TL Metzger KA Gilbert SL. X-ray reconstruction of moving morphology (XROMM): precision, accuracy and applications in comparative biomechanics research. J Exp Zool A Ecol Genet Physiol. (2010) 5:262–79. 10.1002/jez.589 20095029

[20] Haussler KK Bertram JE Gellman K Hermanson JW. Segmental in vivo vertebral kinematics at the walk, trot and canter: a preliminary study. Equine Vet J Suppl. (2001) 33:160–4. 10.1111/j.2042-3306.2001.tb05381.x 11721560

[21] Deban SM Schilling N Carrier DR. Activity of extrinsic limb muscles in dogs at walk, trot and gallop. J Exp Biol. (2012) 215:287–300. 10.1242/jeb.063230 22189773

[22] Hildebrand M. Analysis of the symmetrical gaits of tetrapods. Folia biotheoretica. (1966) 6:9–22.

[23] Hildebrand M. Symmetrical gaits of dogs in relation to body build. J Morphol. (1968) 3:353–60. 10.1002/jmor.1051240308 5657937

[24] Fischer MS Lilje KE. Hunde in Bewegung. Stuttgart: Franckh-Kosmos-Verlag (2011). p. 34.

[25] Gregory CR Cullen JM Pool R Vasseur PB. The canine sacroiliac joint. Preliminary study of anatomy, histopathology, and biomechanics. Spine. (1976) 10:1044–8. 10.1097/00007632-198612000-00019 3576343

[26] Wennerstrand J. Clinical perspectives on equine back kinematics (doctoral thesis). Department of Anatomy, Physiology and Biochemistry, Swedish University of Agricultural Sciences, Uppsala, Sweden (2008).

[27] Faber M Schamhardt H van Weeren R Johnston C Roepstorff L Barneveld A. Basic three-dimensional kinematics of the vertebral column of horses walking on a treadmill. Am J Vet Res. (2000) 4:399–406. 10.2460/ajvr.2000.61.399 10772104

[28] Faber M Johnston C Schamhardt H van Weeren R Roepstorff L Barneveld A. Basic three-dimensional kinematics of the vertebral column of horses trotting on a treadmill. Am J Vet Res. (2001) 5:757–64. 10.2460/ajvr.2001.62.757 11341399

[29] Wachs K. Kinematische Analyse von 3D-rekonstruierten Bewegungen der Lendenwirbelsäule und des Beckens beim Beagle in Schritt und Trab (doctoral thesis). Tierärztliche Hochschule Hannover, Hanover, Germany (2015).

[30] Schilling N Carrier DR. Function of the epaxial muscles during trotting. J Exp Biol. (2009) 7:1053–63. 10.1242/jeb.020248 19282502

[31] Fischer S Nolte I Schilling N. Adaptations in muscle activity to induced, short-term hindlimb lameness in trotting dogs. PLoS ONE. (2013) 8:11. 10.1371/journal.pone.0080987 24236207 PMC3827467

[32] Nunamaker DM Blauner PD. Normal and abnormal gait. In: Newton CD, Nunamaker DM, editors. Textbook of Small Animal Orthopaedics. Philadelphia, PA: JP Lippincott Company (1985). p. 1083–95.

[33] DeCamp CE. Kinetic and kinematic gait analysis and the assessment of lameness in the dog. Vet Clin North Am Small Anim Pract. (1997) 4:825–40. 10.1016/S0195-5616(97)50082-9 9243783

[34] Braund KG Taylor TK Ghosh P Sherwood AA. Spinal mobility in the dog. A study in chondrodystrophoid and non-chondrodystrophoid animals. Res Vet Sci. (1977) 1:78–82. 10.1016/S0034-5288(18)33317-4 841208

[35] Adams MA Roughley PJ. What is intervertebral disc degeneration, and what causes it? Spine. (2006) 18:2151–61. 10.1097/01.brs.0000231761.73859.2c 16915105

[36] Krismer M Haid C Ogon M Behensky H Wimmer C. Biomechanik der lumbalen Instabilität [Biomechanics of lumbar instability]. Orthopade. (1997) 6:516–20. 10.1007/PL00003406 9333739

[37] Adams MA Hutton WC. The mechanical function of the lumbar apophyseal joints. Spine. (1983) 3:327–30. 10.1097/00007632-198304000-00017 6623200

[38] Cyron BM Hutton WC Stott JR. Spondylolysis: the shearing stiffness of the lumbar intervertebral joint. Acta Orthop Belg. (1979) 4:459–69. 534378

[39] Schilling N Carrier DR. Function of the epaxial muscles in walking, trotting and galloping dogs: implications for the evolution of epaxial muscle function in tetrapods. J Exp Biol. (2010) 9:1490–502. 10.1242/jeb.039487 20400634

[40] Kirkaldy-Willis WH. The relationship of structural pathology to the nerve root. Spine. (1984) 1:49–52. 10.1097/00007632-198401000-00010 6719256

[41] Sukthankar A Nerlich AG Paesold G. Age-related changes of the spine. In: Boos N, Aebi M, editors. Spinal Disorders. Heidelberg: Springer (2008). p. 91–122.

[42] Kopp KI. Kinematik des Beckens und der kaudalen Lendenwirbelsäule beim Deutschen Schäferhund: eine Untersuchung mittels biplanarer Röntgenvideographie und Scientific Rotoscoping (doctoral thesis). Justus Liebig University of Giessen, Giessen, Germany (2019).

