# Supplementary material for: Three-Dimensional Kinematics of the Pelvis and Caudal Lumbar Spine in German Shepherd Dogs
Source: Front Vet Sci. 2021 Aug 26;8:709966. doi: 10.3389/fvets.2021.709966 (PMC8427507; doi:10.3389/fvets.2021.709966)
Supplement: Supplementary file 5 [file Data_Sheet_1.docx]

Supplementary Material

# Supplementary Data

**X-ray videos are submitted**

Video 1, 2 ,3 and 4

# Supplementary Tables

Table 1: Mean ± standard deviations (Mean ± SD in %) of the timing of the directional changes in rotation. The TOO (time of occurrence) represents the timing of the maxima and minima in % of the stride cycle. TOO was analyzed in all four dogs during walking (n = 6) and trotting (n = 9). The stride cycle started with the left hindlimb (reference leg) with a duty factor of 0.7 for walking and 0.4 for trotting (except GSD 3: 0.5).

Whereas axial and sagittal pelvic rotation was monophasic with two directional changes in rotation per stride cycle (one maximum and one minimum), sagittal pelvic rotation was biphasic (four directional changes in rotation with two maxima and two minima). Axial and lateral rotation of L6 and L7 mainly was bi- to triphasic with a high standard deviation, as this motion was largely asynchronous with the stride cycle.

| pelvis |  |  | dog 1 | dog 2 | dog 3 | dog 4 | all dogs |
| --- | --- | --- | --- | --- | --- | --- | --- |
|  | walk | rx | 20,5±2,6  78,7±1,4 | 28,3±4,3  101,5±7,1 | 41,5±7,0  83,5±5,4 | 25,6±6,5  83,5±11,8 | 29,0±9,0  86,8±10,1 |
|  |  | ry | 37,5±6,7  94,0±3,5 | 45,3±8,2  102,2±4,0 | 44±2,0  95±4,1 | 46,2±4,4  93,4±1,5 | 43,3±3,9  96,2±4,0 |
|  |  | rz | 3,7±3,1  27,7±5,2  58,8±4,0  81,5±2,3 | 4,5±3,8  29,3±6,1  56,7±1,9  85,0±5,4 | 1,4±2,6  32,4±4,1  46,8±2,5  78,4±6,5 | 1,8±4,1  32,8±4,5  54,8±3,8  76,8±2,7 | 2,8±1,5  30,6±2,5  54,3±5,2  80,4±3,6 |
|  | trot | rx | 25,8±3,5  74,9±7,1 | 35,3±3,1  109,7±2,3 | 47,2±8,3  98,4±11,5 | 23,7±5,8  71,0±6,2 | 33±10,7  88,5±18,6 |
|  |  | ry | 41,1±4,2  90,4±2,7 | 34,8±8,8  87,9±4,0 | 54,2±4,9  104,0±3,4 | 29,8±5,5  85,5±10,3 | 39,9±10,6  91,9±8,3 |
|  |  | rz | 20,2±1,5  45,7±1,5  77,8±2,7  98,8±2,1 | 24,5±4,2  44,0±9,0  71,6±0,9  97,1±3,1 | 34,5±7,1  58,6±10,3  81,9±13,5  101,8±6,6 | 18,3±3,4  42,9±5,1  67,9±3,0  94,3±5,2 | 24,4±7,2  47,8±7,3  74,8±6,3  98,0±3,1 |
| L7 | walk | rx | 5,2±6,2  44,0±3,7  74,8±6,6  22,3±2,2  58,0±5,8  88,2±4,5 | 13,2±5,7  54,3±3,2  77,8±6,2  35,6±6,4  65,0±6,6  101,8±10,6 | 2,2±10,9  35,3±13,7  76,5±7,2  18,0±11,5  57,2±9,9  87,7±8,6 | 4,8±5,8  36,2±17,6  79,2±12,1  19,0±10,7  59,0±14,7  95,2±10,0 | 6,3±4,8  42,5±8,8  77,1±1,9  23,7±8,1  59,8±3,6  93,2±6,7 |
|  |  | ry | 42,0±17,4  62,0±7,2  94,5±4,1  17,8±10,8  58,8±16,8  - | 37,7±35,8  50,3±25,5  80,4±15,3  4,8±19,5  36,0±22,7  60,0±20,8 | 10,8±15,7  47,0±10,7  82,0±9,9  30,3±9,3  63,8±9,6  87,0±9,7 | 18,2±4,7  51,4±3,5  87,2±7,7  1,5±6,8  40,3±10,0  70,8±4,7 | 27,2±15,0  52,7±6,5  86,0±6,4  13,6±13,2  49,7±13,6  72,6±13,6 |
|  |  | rz | 2,5±3,3  41,8±6,6  -  24,3±5,1  65,3±13,2  - | 10,3±8,1  38,4±15,3  72,3±13,0  28,8±13,7  59,0±17,5  90,2±8,9 | 18,0±9,9  51,0±14,6  85,0±8,3  5,5±9,4  36,5±10,2  65,8±12,9 | 9,5±8,0  40,8±13,8  71,2±21,2  24,3±13,2  61,3±21,0  79,8±17,9 | 10,1±6,3  43,0±5,5  76,2±7,7  20,8±10,4  55,5±13,0  78,6±12,2 |
|  | trot | rx | 70,6±7,3  -  97,0±6,9  24,8±5,0  53,1±10,7  - | 25,7±7,5  53,1±7,3  88,9±14,7  16,0±5,9  36,3±10,3  82,4±12,4 | 13,0±8,9  65,1±8,1  -  31,1±9,1  -  92,1±8,5 | 47,3±10,2  77,3±12,9  -  29,0±13,9  -  93,2±16,5 | 39,1±25,3  73,1±18,7  93,0±5,7  32,4±15,4  44,7±11,9  89,2±5,9 |
|  |  | ry | 8,8±20,3  34,3±5,6  63,5±3,5  43,0±27,6  54,5±13,4  94,5±0,7 | 24,2±23,8  -  65,3±24,7  43,5±24,2  -  99,2±14,3 | 15,9±18,6  -  63,0±17,6  39,8±22,9  -  84,9±16,5 | 13,4±21,2  -  71,1±16,6  40,7±12,9  -  - | 15,6±6,4  -  65,7±3,7  41,7±1,8  -  94,5±7,3 |
|  |  | rz | 26,1±7,2  82,9±5,3  8,9±2,4  53,6±6,4 | 28,8±2,8  83,9±4,3  7,9±5,3  59,4±9,8 | 38,4±4,5  85,7±4,9  11,1±8,3  62,4±4,5 | 26,6±5,4  82,7±5,9  10,6±1,5  55,3±9,0 | 30,0±5,7  83,8±1,4  9,6±1,5  57,7±4,0 |
| L6 | walk | rx | 26,5±8,3  71,0±11,8  85,5±0,7  4,7±9,7  50,5±8,5  79,0±6,0 | 44,5±41,0  55,7±14,0  80,0±6,7  1,0±12,1  38,7±16,0  68,8±11,3 | 13,7±8,1  46,2±4,9  87,0±9,0  2,3±6,8  30,5±6,4  62,7±10,9 | 16,8±10,9  55,0±9,8  82,0±7,0  3,0±7,8  36,3±15,0  64,3±9,7 | 25,4±13,9  57,0±10,3  83,6±3,2  2,8±1,5  39,0±8,4  68,7±7,3 |
|  |  | ry | 20,3±5,5  54,0±9,8  78,4±6,5  40,0±8,5  62,8±10,0  96,5±8,9 | 13,2±9,5  37,5±6,5  80,0±9,4  25,0±4,6  57,0±7,3  100,0±7,3 | 31,0±10,6  59,5±2,6  88,0±9,1  5,6±6,8  47,8±12,2  93,7±9,6 | 3,3±1,5  41,3±14,9  80,5±8,7  20,8±3,9  61,8±11,9  98,2±10,3 | 17,0±11,7  48,1±10,4  81,8±4,3  22,8±14,2  57,4±6,8  97,1±2,7 |
|  |  | rz | 16,3±13,4  59,5±9,9  85,3±4,5  2,8±7,8  39,8±11,1  74,5±9,3 | 18,5±7,2  52,8±11,1  83,5±1,7  -1±4,7  35,5±12,1  66,5±4,9 | 18,8±9,0  48,7±18,5  72,6±10,3  4,3±8,6  29,8±9,1  53,2±9,7 | 8,2±4,7  44,4±21,4  85,5±11,1  29,5±14,7  64,3±13,9  99,8±7,3 | 15,5±5,0  51,4±6,4  81,7±6,2  8,9±13,9  42,4±15,2  73,5±19,6 |
|  | trot | rx | 22,1±15,8  65,0±17,1  84,0±2  5,3±8,5  44,2±14,5  77,3±12,8 | 21,8±14,9  65,6±17,6  90,8±11,0  10,0±11,3  41,2±9,7  60,8±42,2 | 10,8±13,3  43,5±14,6  75,3±11,6  26,4±16,6  58,9±13,0  94,4±14,3 | 30,0±21,2  85,2±10,1  -  63,7±23,1  23,6±8,7  - | 21,2±7,9  64,8±17,0  83,4±7,8  26,4±26,5  42,1±14,5  77,5±16,8 |
|  |  | ry | 18,0±8,0  71,0±13,4  -  36,3±8,8  84,5±17,8  - | 17,8±6,3  52,0±9,9  85,5±5,9  6,7±13,0  49,1±15,4  77,7±13,0 | 19,9±10,8  65,8±10,9  -  45,1±13,4  87,9±16,8  - | 32,0±14,2  83,6±10,8  -  9,8±18,2  60,8±9,6  - | 21,9±6,8  68,1±13,1  -  24,5±19,1  70,6±18,7  - |
|  |  | rz | 23,6±16,7  80,8±8,5  28,0±15,5  72,1±20,7 | 30,2±9,0  70,1±11,3  34,8±16,0  84,2±13,1 | 18,6±9,4  64,6±9,0  41,8±10,8  85,5±18,8 | 17,2±9,8  67,3±11,3  40,8±8,7  94,0±11,3 | 22,4±5,9  70,7±7,1  36,3±6,4  80,3±10,9 |

dard deviations (Mean ± SD in °) of the range of motion (ROM). ROM was analyzed in all four dogs during walking (n = 6) and trotting (n = 9). Values with a grey background present the main direction of movement for the pelvis, L6, and L7.

|  |  |  |  | dog 1 | dog 2 | dog 3 | dog 4 | all dogs |
| --- | --- | --- | --- | --- | --- | --- | --- | --- |
| **ROM (°)** | pelvis | walk | rx | 18,9±2,8 | 10,8±3,7 | 11,4±0,7 | 7,8±2,8 | **12,1±4,7** |
|  |  |  | ry | 10,2±1,3 | 11,1±3,4 | 10,3±2,7 | 12,0±4,3 | **10,9±0,83** |
|  |  |  | rz | 5,1±0,8 | 7,1±0,9 | 7,8±0,7 | 11,2±2,6 | **7,8±2,5** |
|  |  | trot | rx | 8,0±0,6 | 5,9±0,8 | 11,0±1,6 | 7,5±2,2 | **6,1±5,7** |
|  |  |  | ry | 8,7±2,5 | 9,6±1,5 | 9,8±2,6 | 7,8±1,8 | **9,0±0,9** |
|  |  |  | rz | 8,7±2,5 | 7,7±2,5 | 4,2±0,6 | 6,2±1,7 | **6,7±1,9** |
|  |  |  |  |  |  |  |  |  |
|  | L7 | walk | rx | 2,9±0,3 | 2,8±0,8 | 3,7±1,0 | 2,7±0,7 | **3,0±0,5** |
|  |  |  | ry | 3,8±0,5 | 3,2±0,7 | 4,3±1,1 | 3,2±0,7 | **3,6±0,6** |
|  |  |  | rz | 3,2±0,4 | 2,9±0,4 | 4,0±0,5 | 4,3±1,5 | **3,6±0,7** |
|  |  | trot | rx | 3,4±0,8 | 2,9±0,6 | 2,8±0,6 | 3,0±1,0 | **3,0±0,3** |
|  |  |  | ry | 3,7±0,5 | 2,6±0,5 | 4,7±0,9 | 2,7±0,6 | **3,4±1,0** |
|  |  |  | rz | 4,9±1,0 | 5,7±1,3 | 4,6±1,0 | 5,1±1,8 | **5,1±0,5** |
|  |  |  |  |  |  |  |  |  |
|  | L6 | walk | rx | 2,5±0,6 | 2,0±0,5 | 2,9±0,5 | 2,6±0,7 | **2,5±0,4** |
|  |  |  | ry | 4,4±0,9 | 3,5±0,9 | 3,3±0,8 | 3,9±1,1 | **3,8±0,5** |
|  |  |  | rz | 3,2±0,5 | 2,3±0,4 | 4,3±0,6 | 2,5±0,5 | **3,1±0,9** |
|  |  | trot | rx | 2,1±0,5 | 2,1±0,5 | 2,2±0,4 | 2,4±0,6 | **2,3±0,2** |
|  |  |  | ry | 4,4±0,8 | 3,6±0,8 | 4,0±0,8 | 3,4±0,6 | **3,8±0,4** |
|  |  |  | rz | 3,7±1,0 | 3,1±0,6 | 3,5±0,6 | 3,1±0,6 | **3,3±0,3** |
|  |  |  |  |  |  |  |  |  |

|  |  |  |  | dog 1 | dog 2 | dog 3 | dog 4 | all dogs |
| --- | --- | --- | --- | --- | --- | --- | --- | --- |
| **ROM(cm)** | pelvis | walk | tx | 2,0±0,5 | 2,4±0,5 | 2,7±0,8 | 3,4±1,3 | **2,6±0,6** |
|  |  |  | ty | 2,7±0,4 | 3,7±0,3 | 3,5±0,5 | 4,6±0,8 | **3,6±0,8** |
|  |  |  | tz | 1,9±0,5 | 5,4±2,8 | 2,7±0,8 | 4,1±5,2 | **3,5±1,6** |
|  |  | trot | tx | 2,5±1,2 | 2,3±0,8 | 2,7±0,6 | 3,4±1,9 | **2,9±0,5** |
|  |  |  | ty | 3,9±0,5 | 3,5±0,5 | 2,8±0,3 | 4,1±0,6 | **3,6±0,6** |
|  |  |  | tz | 1,6±0,4 | 3,3±1,0 | 2,9±1,1 | 2,6±1,1 | **2,6±0,7** |
|  |  |  |  |  |  |  |  |  |
|  | L7 | walk | tx | 0,14±0,03 | 0,12±0,02 | 0,20±0,04 | 0,15±0,04 | **0,15±0,03** |
|  |  |  | ty | 0,16±0,04 | 0,21±0,04 | 0,20±0,04 | 0,20±0,06 | **0,19±0,02** |
|  |  |  | tz | 0,22±0,08 | 0,26±0,09 | 0,20±0,08 | 0,29±0,12 | **0,24±0,04** |
|  |  | trot | tx | 0,15±0,05 | 0,26±0,07 | 0,17±0,03 | 0,19±0,05 | **0,19±0,05** |
|  |  |  | ty | 0,17±0,06 | 0,18±0,07 | 0,15±0,05 | 0,20±0,06 | **0,17±0,02** |
|  |  |  | tz | 0,19±0,04 | 0,20±0,09 | 0,15±0,03 | 0,20±0,06 | **0,18±0,02** |
|  |  |  |  |  |  |  |  |  |
|  | L6 | walk | tx | 0,09±0,02 | 0,12±0,04 | 0,16±0,02 | 0,12±0,04 | **0,12±0,03** |
|  |  |  | ty | 0,16±0,04 | 0,15±0,08 | 0,20±0,04 | 0,21±0,04 | **0,18±0,03** |
|  |  |  | tz | 0,20±0,08 | 0,15±0,06 | 0,14±0,04 | 0,22±0,08 | **0,18±0,04** |
|  |  | trot | tx | 0,13±0,04 | 0,15±0,03 | 0,15±0,04 | 0,11±0,03 | **0,13±0,02** |
|  |  |  | ty | 0,13±0,07 | 0,13±0,06 | 0,08±0,02 | 0,21±0,06 | **0,14±0,05** |
|  |  |  | tz | 0,10±0,07 | 0,14±0,07 | 0,02±0,01 | 0,23±0,07 | **0,12±0,09** |
|  |  |  |  |  |  |  |  |  |
